# Strain Specific Genotype−Environment Interactions and Evolutionary Potential for Body Mass in Brook Charr (*Salvelinus fontinalis*)

**DOI:** 10.1534/g3.112.005017

**Published:** 2013-03-01

**Authors:** Amélie Crespel, Louis Bernatchez, Céline Audet, Dany Garant

**Affiliations:** * Institut des Sciences de la Mer de Rimouski, Université du Québec à Rimouski, Rimouski, QC, G5L 3A1, Canada; † Institut de Biologie Intégrative et des Systèmes, Département de biologie, Université Laval, Québec City, QC, G1V 0A6, Canada; ‡ Département de biologie, Université de Sherbrooke, Sherbrooke, QC, J1K 2R1, Canada

**Keywords:** heritability, evolutionary potential, gene−environment interactions, genetic architecture, strains

## Abstract

Discriminating between genetic and environmental causes of phenotypic variation is an essential requirement for understanding the evolutionary potential of populations. However, the extent to which genetic variation differs among conspecific groups and environments during ontogeny has rarely been investigated. In this study, the genetic basis of body mass was measured in three divergent strains of brook charr (*Salvelinus fontinalis*) in different rearing environments and at different time periods. The results indicate that body mass was a heritable trait in all strains but that the level of heritability greatly differed among strains. Moreover, heritability estimates of each strain varied differently according to environmental rearing conditions, and cross-environments correlations were all significantly lower than unity, indicating strain-specific patterns of genotype–environment interactions. Heritability estimates also varied throughout ontogeny and decreased by 50% from 9 to 21 months of age. This study highlights the divergence in genetic architecture and evolutionary potential among these strains and emphasizes the importance of considering the strain-specific potential of the response to selection according to environmental variation.

In the current context of increasing anthropogenic selection pressures, such as climate change or artificial selection through exploitation, it is becoming increasingly important to document the evolutionary potential of populations (Smith and Bernatchez 2008; Visser 2008), *i.e.*, their capacity to adapt to changing environmental conditions in the long term. To this end, one needs to assess the evolutionary and plastic environmental components underlying phenotypic variation, which is best achieved using a quantitative genetics approach. For instance a low additive genetic variance and low heritability will reduce the capacity of populations to respond to selection and would be a major obstacle for evolution (Lynch and Walsh 1998). The amount of additive genetic variance usually differs among populations according to local adaptations to native environments (*e.g.*, Laugen *et al.* 2005; DiBattista *et al.* 2008; Visscher *et al.* 2008); in addition, genetic variance of a trait within a population can also vary depending on environmental conditions (Hoffmann and Merilä 1999; Charmantier and Garant 2005). Most evidence gathered so far suggests a greater heritability of morphometric traits under favorable conditions in wild species (see Wilson *et al.* 2006, for example; reviewed in Charmantier and Garant 2005).

A plastic response to environmental effects could also have a heritable genetic basis and be itself adaptive, resulting in genotype−environment interactions (Nussey *et al.* 2007). Depending on the nature of genotype−environment interactions, they could either facilitate or dampen the populations’ capacity to adapt. The ability of organisms to express different genes under different environmental conditions allows the maintenance of additive genetic variance, but plastic response may also counteract response to selection and thus reduce the speed of evolutionary changes (Wilson *et al.* 2006), which may be consequential in applied contexts as well. In fish culture, for example, the occurrence of genotype−environment interactions are frequently investigated because they may hamper the diffusion of genetic gain in different rearing environments and thus be undesirable (Fishback *et al.* 2002; Kause *et al.* 2004; Maluwa *et al.* 2006; Saillant *et al.* 2006). Different populations of the same species may also express different sensitivities to environmental variations (*e.g.*, Falconer and Mackay 1996; Laurila *et al.* 2002; Husby *et al.* 2010), but until now very few studies have sought to compare genotype-environment interaction among populations.

The occurrence of parental effects will also modify the offspring’s genetic basis and phenotypic expression. Maternal effects are generally detected during early life, when maternal genotype or phenotype (such as maternal care, maternal feeding, or yolk sac quality and quantity) influences offspring development (Falconer and Mackay 1996; Heath *et al.* 1999; Perry *et al.* 2004). Paternal effects can also modify offspring phenotypes, but the underlying mechanisms are still unclear (*e.g.*, Pakkasmaa *et al.* 2001; Garant *et al.* 2003; Serbezov *et al.* 2010). Parental effects may vary among populations and have consequences on the divergence of adaptive evolutionary responses (Perry *et al.* 2005; Kruuk *et al.* 2008). Finally, the genetic basis of traits can also be affected by the developmental stage due to the differential age-specific expression of genes (Atchley and Zhu 1997; Wilson and Réale 2006). It is therefore important to examine heritability variations during ontogeny to provide a better understanding of the response to selection throughout lifetime (Wilson and Réale 2006; Robinson *et al.* 2009).

The main objective of this study was to investigate ontogenic change in genetic, environmental, and genotype–environment components influencing body mass among conspecific strains of brook charr (*Salvelinus fontinalis*) to assess their evolutionary potential for this trait. Brook charr is a broadly distributed salmonid that is endemic fish to northeastern North America. Sexual maturation begins early in the summer, reproduction occurs in the fall, and embryos will develop at low temperatures during winter time. At hatching, yolk-sac fry will depend on nutriments stored in the eggs until they reach the swim-up stage and feed on external sources. The duration of the juvenile growing stage will be very variable among populations. In captivity, sexual maturation can occur as early as 0+ males and 1+ females based on spawning time and growth boost obtained through temperature and feeding conditions, but in the wild, growth cannot be sustained at such a rate and the juvenile stage will be very much longer. Since its postglacial colonization of eastern Canada that occurred about 11,000 years ago (Castric and Bernatchez 2003; Fraser and Bernatchez 2005c), the species has occupied various environments. Thus, brook charr can be either lacustrine (*e.g.*, Fraser and Bernatchez 2005b), river-resident, or anadromous, inhabiting fresh or brackish water (*e.g.*, Curry *et al.* 2010). It can also adapt to artificial environments and is an economically important species that represents 60% of Québec’s freshwater aquaculture production (MAPAQ 2007). Understanding the evolutionary potential of brook charr is thus of fundamental interest both because it can be sensitive to various conditions and because of implications for its management in aquaculture.

In teleost fishes, body mass is related to different components of fitness such as survival, life history tactic, or reproductive success (Sogard 1997; Wilson *et al.* 2003; Garcia de Leaniz *et al.* 2007; Thériault *et al.* 2007; Serbezov *et al.* 2010; Varian and Nichols 2010), and thus it can be considered as one of the most important fitness-related traits. In this context, our specific objectives were (1) to investigate differences in the genetic basis of body mass among the three strains of brook charr by comparing the relative importance of additive genetic effects in a common environment, (2) to estimate the importance of genotype−environment interaction in the genetic control of body mass, and (3) to assess the parental and ontogenic effects on the observed patterns.

## Materials and Methods

### Brook charr strains

The Laval strain (L) originates from a wild anadromous population from the Laval River (48°44′N; 69°05′W) on the north shore of the St. Lawrence estuary (QC, Canada). The fish used were from third-generation breeders reared in captivity at the Station aquicole Institut des sciences de la mer de Rimouski (ISMER)/Université du Québec à Rimouski (Rimouski, QC, Canada). The Rupert strain (R) originates from a northern lacustrine freshwater-resident wild population inhabiting the Rupert River system (51°05′N; 73°41′W; QC, Canada). The fish used as breeders were also from the third generation produced in captivity at the Laboratoire régional en sciences aquatiques (LARSA, Laval University, Québec, QC, Canada). The domestic strain (D) has been widely used by Québec’s fish farming industry for more than a hundred years and originates from many crosses between two freshwater strains (Nashua and Baldwin). Breeders of the domestic strain were obtained from the Pisciculture de la Jacques Cartier (Cap-Santé, QC, Canada). On the basis of estimates of Shriver *et al.* (1995) using microsatellite data, these three strains were highly differentiated; L and R strains were separated by 13.3 Dsw (genetic distance), with the D strain being about equally genetically distant (about 6.7 Dsw) from the two others (Martin *et al.* 1997). Moreover, 76.2% of the alleles from the wild origin populations were not found in the D population, resulting in high F_ST_ between the D *vs.* R and L populations (mean ± SD, F_ST_ = 0.187 ± 0.009). The L and R populations were even more genetically differentiated than the D *vs.* L or D *vs.* R populations (mean F_ST_ = 0.427 ± 0.020. Finally, Martin *et al.* (1997) found no evidence for increased inbreeding in any of these three populations that had inbreeding coefficient (F_IS_) values varying between 0.18 and 0.35, which were similar to values typically observed for wild brook charr populations (Fraser and Bernatchez 2005a; Marie *et al.* 2010). The three strains also differ importantly in regulation of gene expression when reared in common environments (Bougas *et al.* 2010).

### Family crosses and rearing

Three purebred cross-types (D_♀_D_♂_, L_♀_L_♂_, and R_♀_R_♂_) were made from mid-November 2005 until the end of December 2005 at LARSA between 10 sires and 10 dams of each strain (Supporting Information, Table S3). All breeders were used only once, and 10 full-sib families were obtained from each cross. During the first six months, *i.e.*, from egg incubation (January) until exogenous feeding (June), families were kept separate in recirculating freshwater and reared in seven troughs, each of which was divided into 12 units. Water temperature was maintained at 6° during egg incubation and at 8° after hatching. In June, families were marked so as to allow identification by different combinations of adipose and pelvic fin clippings and then transferred to nine 3-m^3^ tanks with eight families per tank. All individuals from a given family were reared together in the same tank. All families were brought to 2136 degree-days by the end of the summer and maintained at 10°. The photoperiod followed the natural seasonal cycle and fish were fed according to commercial charts.

### Rearing environments and body mass measurements

In September 2006, fish from each family were randomly divided and transferred in transport bags (one family per bag) to one of two rearing environments that differed according to tank rearing system, water source, and water temperature conditions. In each environment, all three strains were reared under similar conditions. At ISMER, for each strain, 230 fish per family were reared in ten 0.5-m^3^ indoor tanks, with six to eight families per tank using the initial pool conditions set up at LARSA. Fish were kept under natural temperature (from 3° in winter to 15° in summer) and photoperiod conditions (Table S4) in running dechlorinated fresh water (fish density about 35 kg m^3^). To maintain sustainable rearing densities, the number of fish per family was gradually reduced to 60 by the end of the experiment, with all reductions in number being done randomly. Fish were fed daily (1% w/w ration) with commercial dry pellets. At LARSA, for each strain, 150 fish per family were reared in nine 3-m^3^ indoor tanks under natural photoperiod conditions at constant temperature of 10° in recirculating fresh water (fish density about 20 kg m^3^). Fish numbers were gradually decreased to 50 fish per family by the end of the experiment. Fish were fed daily (1% w/w ration) with commercial dry pellets. This experiment ended in November 2007, when fish were 21 months old. The first three samplings were made at LARSA: 20 fish par family were randomly sampled (n = 600) when yolk-sac resorption was complete (about two months old), 50 fish per family were randomly sampled (n = 1500) at 15 weeks after exogenous feeding began (about 4 months old), and 50 fish per family were randomly sampled (n = 1500) at 2136 degree-days in September 2006 (about 7 months old). After transfer to the two rearing locations, 25 fish per family (n = 750 for each location) were randomly sampled every eight weeks. For each sampling, fish were anesthetized in MS 222 (0.16 g L^-1^ [3-aminobenzoic acid ethyl ester]) and their body mass (to the nearest 0.1 g) was measured. Body mass was also recorded for every remaining fish at the final sampling in November 2007 (Table S2).

### Data analysis

Data normality and homogeneity of variance were tested with Kolmogorov-Smirnov and the Brown-Forsythe tests, respectively. Body mass data were log-transformed before analysis to obtain normality and account for heteroscedasticity. Variance components were analyzed separately in each strain and environment for each sampling time (ontogeny) and were estimated by restricted maximum likelihood implemented in ASReml (V2.0) (Gilmour *et al.* 2006) using the following model:

y=µ+A+e

where *y* is the phenotypic observation; *µ* is the overall mean; *A* is the additive genetic effect linked to the pedigree structure (full-sib families); and *e* is the residual. The total phenotypic variance (V_P_) of each trait was decomposed into the additive genetic variance (V_A_) and the residual variance (V_R_). The broad-sense heritability (*h*^2^) for each trait was estimated as the ratio of the estimated additive genetic variance to the total phenotypic variance: *h*^2^ = V_A_/V_P_. Genetic correlations between ISMER and LARSA were also estimated for each strain using a bivariate model in which phenotypic observations at the two sites were included, sampling time (from 9 to 21 months of age), being considered as a fixed effect: *r*_G_ = COV_Ai,j_/(V_Ai_ × V_Aj_). SEs for variance and covariance components as well as for heritabilities and genetic correlations were also estimated using ASReml. The statistical significance of the additive genetic variance and covariance were tested by comparing the full model with a restricted model, where the additive variance (or covariance) was set to zero (and also to unity for estimation of genetic correlations between environments), using a likelihood ratio test (against the χ^2^ distribution, where χ^2^ = −2*difference in log likelihood). The relative influence of maternal *vs.* paternal effect on progeny mass in each strain, and at each time sampling, was estimated as described by Heath *et al.* (1999) which is a regression of mean offspring size against the size of each of their parents. The difference between the two regression slopes indicated the relative influence of maternal or paternal effects. Maternal effects were considered to be present when the relative influence was significantly positive while a negative relative influence indicated that variations in the progeny were more related to sire effects (Heath *et al.* 1999).

Statistical analysis on the complete interaction (strain × environment × time) was not possible because only one measure of heritability for each data set was obtained. Therefore, we analyzed the effects of strains on heritability estimates and parental effects with randomized block analyses of variance (ANOVAs) and the significance of differences among heritability estimates according to environments was analyzed using two-way ANOVAs including strain, rearing environment, and strain × rearing environment interaction as factors. The change in heritability estimates through time was then analyzed using Spearman correlations. *A posteriori* Tukey tests were used for mean comparisons. Statistical analyses were conducted using Statistica version 6.0 for Windows (StatSoft Inc, Tulsa, OK).

## Results

### Additive genetic effects

**Figure 1:**
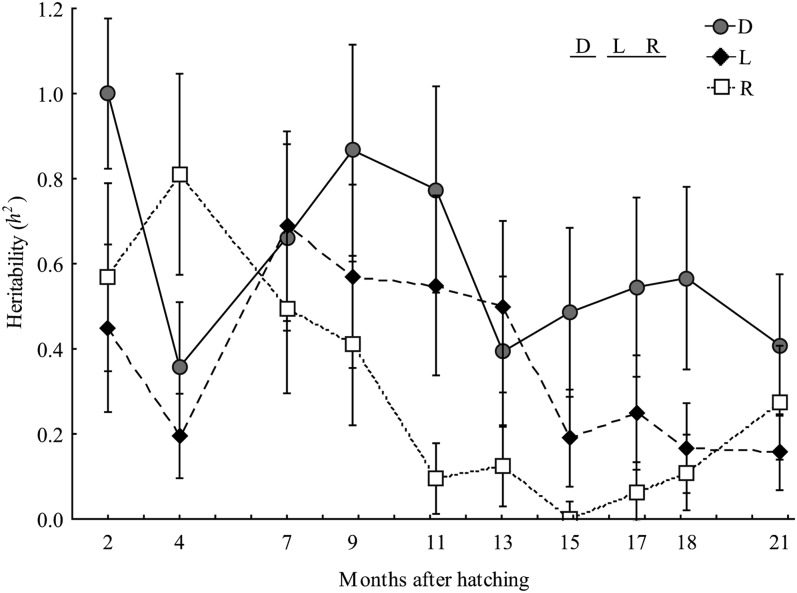
Temporal variation in heritability estimates. Heritabilities (*h^2^* ± SE) for body mass were estimated from yolk sac resorption to 21 months of age for the three brook charr strains (domestic [D], Laval [L], Rupert [R]) reared in the constant temperature environment (LARSA). The statistical difference among the three strains was assessed using randomized block ANOVAs. Results are indicated in the top right corner; letters sharing the same underline are not statistically different.

For the constant temperature environment, heritability estimates were generally moderate to high (between 0.20 and 0.80) and differed among strains (Table 1, ANOVA 1; Figure 1). Heritability was significantly greater in the domestic strain (0.61 ± 0.07) than in the two others, which were not different from one another (0.37 ± 0.06 and 0.30 ± 0.08 for the Laval and the Rupert strains, respectively).

**Table 1:** Summary of ANOVAs

ANOVAs	df	Mean squares	F	*P*
ANOVA 1: Strain effect on the heritability of body mass				
Time	9	0.08	2.16	0.08
Strain	2	0.26	6.92	0.01
Error	18	0.04		
ANOVA 2: Strain and environments effects on the heritability of body mass				
Environment	1	0.01	0.23	0.63
Strain	2	0.64	33.62	< 0.001
Environment × strain	2	0.18	9.40	< 0.001
Error	36	0.02		
ANOVA 3: Strain effect on the parental influence on body mass				
Time	9	0.65	3.11	0.02
Strain	2	1.20	5.78	0.01
Error	18	0.21		

ANOVA 1: randomized block ANOVA, strain effect on heritability; ANOVA 2: two-way ANOVA, strain and environmental effects on heritability; ANOVA 3, randomized block ANOVA, strain and ontogenic effects on parental effects. ANOVA, analysis of variance; Df, the degrees of freedom; F, F-ratio; *P*, *P* values.

### Genotype−environment interactions

**Figure 2:**
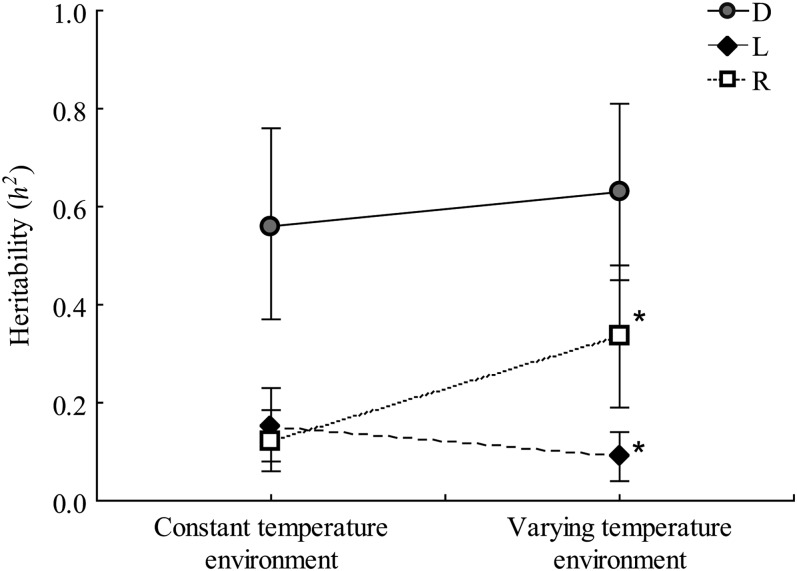
Comparison of heritability among strains and environments. Heritabilities (*h^2^* ± SE) for body mass were estimated for juvenile stages (data on samplings done from 9 to 21 months of age) for the three strains (domestic [D], Laval [L], Rupert [R]) in the constant temperature environment (LARSA) and in the varying temperature environment (ISMER). The statistical difference was assessed using two-way ANOVAs. Asterisks indicate significantly different means between environments (*P* < 0.05).

A significant interaction was observed between strains and environments on heritability estimates (Table 1, ANOVA 2). No significant environmental effect was detected in the domestic strain (*P* = 0.14; Figure 2), whereas we observed a significant genetic covariance and a high correlation between the additive components measured in the two environments (*r*_G_ = 0.87 ± 0.09; Table 2). This genetic correlation was also significantly different from unity (Table 2). The heritability estimates for the Rupert strain were always lower than those of the domestic strain. However, *h^2^* was significantly greater for fish reared in the varying temperature environment (*P* < 0.01) (Figure 2). From 9 to 21 months, the genetic covariance of body mass between the two environments was significant and genetic correlation was high but also significantly different from unity (*r*_G_ = 0.88 ± 0.11; Table 2). For the Laval strain, the *h^2^* estimate was significantly lower in the varying temperature environment relative to the constant temperature environment (*P* = 0.01; Figure 2). In the varying temperature environment, the heritability estimate for the Laval fish was also lower than that of the Rupert strain, and the genetic covariance and the genetic correlation of body mass between the two environments were significantly different from unity but not different from zero (*r*_G_ = 0.50 ± 0.31; Table 2).

**Table 2:** Estimates of correlations between environments

Population	Genetic Correlation	Phenotypic Correlation	Genetic Covariance	*P* Value*s* 0	*P* Value*s* 1
Domestic	0.87 ± 0.09	0.21 ± 0.11	0.013 ± 0.006	<0.001	<0.001
Laval	0.50 ± 0.31	−0.16 ± 0.03	0.001 ± 0.001	0.209	<0.001
Rupert	0.88 ± 0.11	−0.08 ± 0.05	0.003 ± 0.002	0.003	<0.001

Additive genetic correlations, phenotypic correlations, and genetic covariance for the three populations for body mass at age between two environments (running freshwater, seasonal temperature variations [ISMER]; recirculating freshwater, constant 10° temperature conditions [LARSA]). Means ± SE “*P* values 0” that are <0.05 indicate that genetic covariance is significantly different from zero; “P values 1” that are <0.05 indicate genetic correlations that are significantly different from unity (likelihood ratio test).

### Parental effects and ontogenic changes

**Figure 3:**
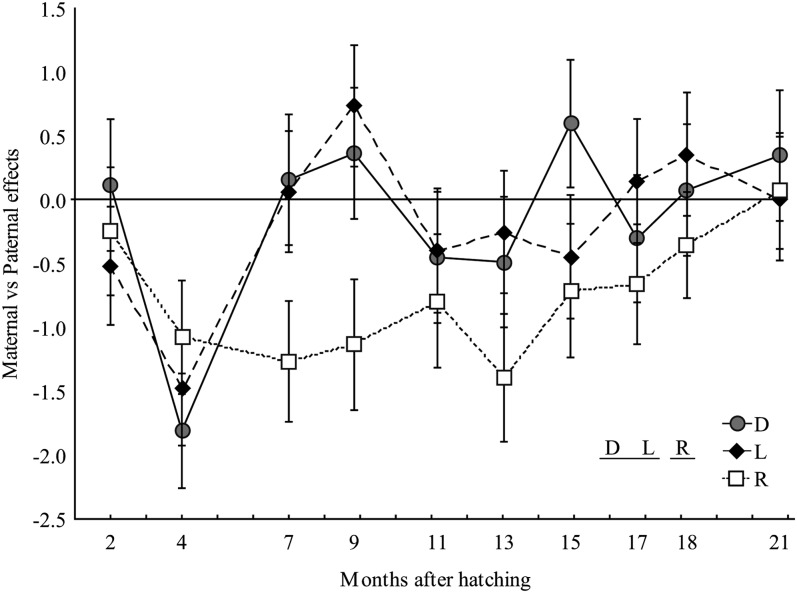
Temporal variation in parental effects. Parental effects (± SE) on body mass were estimated for early developmental (months 2 to 7) and juvenile (months 9 to 21) stages of the three strains (domestic [D], Laval [L], Rupert [R]) in the constant temperature environment (LARSA). Positive values suggest predominantly maternal effects whereas negative values suggest stronger paternal effects. The statistical difference among the three strains was assessed using randomized block ANOVAs. Results are indicated in the bottom right corner; letters sharing the same underline are not statistically different.

Parental effects on progeny mass varied significantly among strains (Table 1, ANOVA 3; Figure 3). Averaged over the time course of the experiment, the relative maternal *vs.* paternal effect was significantly lower in the Rupert strain (−0.75 ± 0.15) than in the two others, which were not different (−0.13 ± 0.21 and −0.17 ± 0.18 for the domestic and the Laval strain, respectively). There was no positive estimate, indicating the absence of maternal effects. However, sire effects were present in the Rupert strain based on significant negative estimates. Finally, during ontogeny, heritability estimates significantly decreased over time (*P* < 0.05; Spearman correlation = −0.70) for both the Laval and the Rupert strains and −0.52 for the domestic strain. The paternal effect on the Rupert strain also decreased from seven to 21 months, changing from being significant to near zero (*P* < 0.05).

## Discussion

The main objective of this study was to compare strain-specific evolutionary potential by documenting differences in genetic, environmental, and genotype-environment components influencing body mass during ontogeny among conspecific strains of brook charr. Heritability estimates for body mass at age were significant in the three strains. However, these estimates differed among strains, with high heritability observed in the domestic strain and moderate heritabilities in the two others. Moreover, rearing environment modified heritability estimates differently according to the strains, and cross-environments correlations were all different from unity revealing strain specific genotype-environment interactions in the genetic control of mass in 9- to 21-month-old fish. Our results thus indicate that these three brook charr strains differ in terms of their evolutionary potential to respond to selection. Finally, heritability also decreased throughout the ontogeny in all three strains, suggesting that the potential to respond to selection may be greater at early in life than later in development.

### Interstrain genetic differences

Heritability of body mass at age was generally very high and almost twofold greater in domestic (0.61 ± 0.07) than in Laval and Rupert strains (0.37 ± 0.06 and 0.30 ± 0.08, respectively). These estimates are of the same order of magnitude as previous estimations of heritability of body mass reported in natural (Thériault *et al.* 2007) and hatchery (Varian and Nichols 2010) brook charr strains. It is true that the full-sib designs used in this study could have resulted in inflated heritability estimates because additive genetic variance cannot be clearly separated from the other variance components related to nonadditive, common environment, or maternal effects (Falconer and Mackay 1996; Merilä *et al.* 2001; Perry *et al.* 2004). However, our results suggest that maternal effects had a limited influence on our estimates. Furthermore, we can assume that, considering the experimental design in common rearing conditions, potential biases were similar among strains and so comparisons among strains and environments are realistic.

A previous study also revealed differences in heritability among two natural populations of brook charr (Wilson *et al.* 2003). However, contrary to our study, these results were obtained under different environmental conditions for each population, such that the observed differences were possibly biased by environmental effects. Our results also corroborate those obtained in other studies performed in common experimental environment that documented the presence of genetic divergence between populations of other vertebrates, particularly in the common frog *Rana temporaria* (Laurila *et al.* 2002; Uller *et al.* 2002; Cano *et al.* 2004) but also the brown trout, *Salmo trutta* (Jensen *et al.* 2008).

Heritability differences among strains indicate divergent genetic architecture and therefore different potentials for evolutionary response to environmental change. In our study, the lower heritability for body mass in the Laval and Rupert strains in different environments suggested that they will respond slower to body mass selection than the domestic strain. Such evidence for quantitative genetic differences between these three strains complement previous studies who reported molecular genetic divergence (Martin *et al.* 1997), as well as genetically based differences in gene regulation between them (Bougas *et al.* 2010). Studying the same families but very early in their development, *i.e.*, at the yolk-sac resorption stage, Bougas *et al.* (2010) performed a transcriptomic analysis by means of microarrays experiments. They showed that the domestic strain was the most differentiated in terms of genome wide patterns, despite the more pronounced genetic distance between the Rupert and Laval strains. Bougas *et al.* (2010) suggested that this could reflect consequences of artificial selection on the domestic strain that occurred through many generations of domestication. This study also revealed differential patterns of gene expression in hybrid crosses between these three populations which indicated that each of them harbors a unique genetic architecture (Bougas *et al.* 2010). In the present study, heritability differences were also due to differences in the amount of additive genetic variance present in each strain (V_A_ around 0.023 for the domestic, 0.012 for the Laval, and 0.009 for the Rupert strains, whereas the phenotypic variance was more similar among strains) (Table S1). Previous studies of genetic diversity in brook charr also reported greater genetic variation at both neutral (Marie *et al.* 2010) and potentially adaptive loci (Lamaze *et al.* 2012b) in domestic fish than in wild populations of brook charr. Wild brook charr populations are often small and isolated from one another which could limit the maintenance of high level of genetic diversity due to a pronounced genetic drift and reduced gene flow, and likely limit the amount of additive variance. On the other hand, domestic brook charr used for aquaculture in Québec may be considered as part of a metapopulation because the management of breeding adults routinely involves fish exchange among different farms. This management activity therefore potentially contributes to maintain genetic diversity by generating gene flow among different rearing sites as well as reducing genetic drift, possibly resulting in maintained additive variance.

### Genotype–environment interactions

We observed marked differences in environmental effects on heritability estimates among strains. Although the domestic strain showed no change relating on the environment, we detected a significant decrease in heritability for the Laval strain and an increase for the Rupert strain between the constant and the varying temperature environment. Under natural conditions, several studies showed that the heritability of morphometric traits of several vertebrates decreases under unfavorable conditions (reviewed in Charmantier and Garant 2005). Such a decrease is generally hypothesized to be the result of a reduction in additive component and/or an increase in environmental variance (Hoffmann and Merilä 1999; Charmantier and Garant 2005). Although none of the controlled environments in our study could be described as being unfavorable for brook charr, our results nevertheless suggest a strain-specific potential of evolutionary response for body mass to environmental changes.

Genetic correlations between the two rearing environments were significantly different from unity for the three strains, suggesting the expression of different sets of genes under the different environments in the three strains. Previous studies have shown that environment modify gene expression in brook charr (Côté *et al.* 2007) and that such an environmental impact may differ among strains according to their distinct genetic architecture, thus causing strain-specific environmental effects. In our study, genetic correlations for the domestic and the Rupert strains were also very strong and close to unity. This suggests that for these two strains, the expression of body mass across environments was not very plastic. On the contrary, it is noteworthy that no significant genetic correlation between the two rearing environments was found for the Laval strain. In the wild, fish from the Laval strain undergo an annual migration from freshwater in summer and fall to an estuarine habitat in spring–summer, thus encountering very contrasting environments on a yearly cycle (Curry *et al.* 2006), which may favor the propensity to phenotypic plasticity (Lind and Johansson 2007; Páez *et al.* 2008). Overall, our results revealed strong strain specific patterns of genotype–environment interactions, further highlighting the fact that populations of the same species may differ for their potential for adaptive response to environmental changes.

In a context of global changes, our results underline different ways through which brook charr might respond to a new environment. First, the high heritability present in the domestic strain could provide an advantage for this strain as it may evolve faster than the two other strains when exposed to environmental selective constraints. On the other hand, the phenotypic plasticity expressed by the Laval population could also be a major asset in terms of adaptations to new environmental conditions. Whether admixed populations would present an advantage cannot be answered at this stage. However, recent studies on brook charr have shown that selection may favor increased introgression between stocked domestic fish and wild populations at genes involved in important phenotypic traits such as growth and that such introgression also affect levels of gene expression and physiological status, namely condition factor (Lamaze *et al.* 2012a, 2012b). Certainly, further work is needed to highlight how different wild populations could cope with the anticipated climate modifications and how wildlife management practices of wild populations will adjust to these new challenges.

### Parental and ontogenic effects

All the families were surveyed from hatching until the beginning of the present experiment. However, during that period, they were maintained at the same culture site, preventing us from studying genetic × environment at this stage. Nevertheless, at hatching, fry from the Laval families were the heaviest and longest and they were still the longest at the yolk-sac resorption stage (Granier *et al.* 2011). This was probably related to direct maternal effects, the Laval dams being the largest ones among the three strains (Perry *et al.* 2004; Granier *et al.* 2011). This effect disappeared once the fry started to feed and, at the end of their first summer of life, juveniles from Domestic families were the largest, the heaviest and had the highest condition factor (Granier *et al.* 2011). While parental effects, especially maternal ones, can influence the genetic variance of offspring, no significant maternal effect was detected and paternal effects were observed only in the Rupert strain during the period of development covered here. The stronger evidence for paternal effects that we found was somewhat unexpected since maternal effects on the early development of offspring are reported more commonly in salmonid fishes (*e.g.*, Heath *et al.* 1999; Perry *et al.* 2004; Serbezov *et al.* 2010). Previous studies also reported the absence of maternal effects (Varian and Nichols 2010), and others reported the occurrence of paternal effects during early development in salmonids (Pakkasmaa *et al.* 2001; Garant *et al.* 2003). To our knowledge however, our study is one of the few to have documented differences in paternal effects among populations of the same species, making brook charr a particularly interesting species to further investigate the underlying mechanisms of paternal effects.

Finally, ontogeny had an important influence on heritability because it decreased during development. Admittedly, this could reflect greater environmental variance increasing with age rather than a decrease in additive effects (Serbezov *et al.* 2010). This is suggested by an increase of total phenotypic variance with age. However, it is noteworthy that additive variance also generally decreased after 9 months of age. This could be linked to the other variance components (*e.g.*, common environments or nonadditive effects) confounded in heritability estimates that could also vary with time (Su *et al.* 1996; Nielsen *et al.* 2010; Serbezov *et al.* 2010). In salmonids, the decrease in heritability through ontogeny has previously been reported for size-related traits (*e.g.*, body mass and length) in various species (Gjerde *et al.* 1994; Choe and Yamazaki 1998; Garant *et al.* 2003; Páez *et al.* 2010; Serbezov *et al.* 2010). During development, several genes can interact differently with other genes or environments, which can modify the global pattern of genetic expression (Atchley and Zhu 1997; Perry *et al.* 2005; Darias *et al.* 2008). Such modifications can alter the genetic control of a complex trait like body mass, creating age-specific potential responses to selection (Atchley and Zhu 1997; Wilson and Réale 2006; Robinson *et al.* 2009). In terms of implications for breeding practices, the decrease in heritability with age suggests that artificial selection for body mass should be more efficient when performed before fish reach sexual maturation. On the other hand, growth is not the only trait that should be considered as early sexual maturation is seen as a negative trait in commercial production. An early selection conducted in the second fall of life and based on mass in non-sexually maturing fish remains one of the best approaches to be considered.

In conclusion, this study represents to our knowledge one of the few studies conducted on vertebrates (research on the common frog being a notable example) in controlled conditions that has revealed pronounced divergence in genotype−environment interactions among strains of a same species. Our results also emphasize the importance of documenting quantitative genetic parameters through time and in different environments in order to better understand strain-specific evolutionary potential in the face of a changing environment.

## Supplementary Material

Supporting Information
