# Supplementary material for: Strain Specific Genotype−Environment Interactions and Evolutionary Potential for Body Mass in Brook Charr (Salvelinus fontinalis)
Source: G3 (Bethesda). 2013 Mar 1;3(3):379–86. doi: 10.1534/g3.112.005017 (PMC3583447; doi:10.1534/g3.112.005017)
Supplement: Supporting Information [file supp_3_3_379__index.html]

Supporting Information 

# Strain Specific Genotype−Environment Interactions and Evolutionary Potential for Body Mass in Brook Charr (*Salvelinus fontinalis*)

## Supporting Information for Crespel *et al.*, 2013

**Files in this Data Supplement:**

- Supporting Information - Tables S1-S4 (PDF, 126 KB)
- Table S1 - Genetic components of body mass at age (PDF, 72 KB)
- Table S3 - Body mass of breeders (PDF, 70 KB)
- Table S4 - Environmental rearing conditions (PDF, 76 KB)
- Table S2 - Raw data of body mass (.xls, 2 MB)
