# Supplementary material for: Strain Specific Genotype−Environment Interactions and Evolutionary Potential for Body Mass in Brook Charr (Salvelinus fontinalis)
Source: G3 (Bethesda). 2013 Mar 1;3(3):379–86. doi: 10.1534/g3.112.005017 (PMC3583447; doi:10.1534/g3.112.005017)
Supplement: Supporting Information [file supp_3.3.379_005017SI.pdf]

G3 · Genes | Genomes | Genetics

Strain specific genotype-environment interactions and evolutionary potential for body mass in brook charr (*Salvelinus fontinalis*)

Amélie Crespel[*], Louis Bernatchez[§], Céline Audet[*1] and Dany Garant[†]

[*]Institut des Sciences de la Mer de Rimouski, Université du Québec à Rimouski, Rimouski, QC, G5L 3A1 Canada ; [§]Institut de Biologie Intégrative et des Systèmes, Département de biologie, Université Laval, Québec City, QC, G1V 0A6, Canada ; and [†]Département de biologie, Université de Sherbrooke, Sherbrooke, QC, J1K 2R1, Canada

[1]Corresponding author: Céline Audet, Institut des Sciences de la Mer de Rimouski, Université du Québec à Rimouski, 310 allée des Ursulines, Rimouski, Québec, G5L 3A1, Canada.
E-mail: celine_audet@uqar.qc.ca
Tel.: +1 418 723 1986 ext. 1744; Fax: +1 418 724 1842

DOI: 10.1534/g3.112.005017

Table S1 Genetic components of body mass at age. Estimates for the three populations at each age and in both environments (running freshwater, seasonal temperature variations [ISMER]; recirculating freshwater, constant 10°C temperature conditions [LARSA]). Estimates of total phenotypic (V_P), residual (V_R), and additive (V_A) variance components; means ± SE.

Age in	domestic			Laval			Rupert		
months	V_P	V_R	V_A	V_P	V_R	V_A	V_P	V_R	V_A
LARSA									
2	0.004 ± 0.001	0.000 ± 0.000	0.004 ± 0.001	0.003 ± 0.001	0.002 ± 0.001	0.002 ± 0.001	0.014 ± 0.002	0.006 ± 0.002	0.008 ± 0.004
4	0.016 ± 0.002	0.010 ± 0.002	0.006 ± 0.003	0.010 ± 0.001	0.008 ± 0.001	0.002 ± 0.001	0.031 ± 0.006	0.006 ± 0.006	0.025 ± 0.012
7	0.029 ± 0.005	0.010 ± 0.005	0.019 ± 0.009	0.017 ± 0.003	0.005 ± 0.003	0.012 ± 0.006	0.026 ± 0.004	0.013 ± 0.004	0.013 ± 0.007
9	0.045 ± 0.010	0.006 ± 0.010	0.039 ± 0.019	0.026 ± 0.004	0.011 ± 0.004	0.015 ± 0.008	0.033 ± 0.005	0.019 ± 0.005	0.014 ± 0.008
11	0.050 ± 0.010	0.011 ± 0.010	0.039 ± 0.019	0.043 ± 0.007	0.019 ± 0.007	0.023 ± 0.012	0.040 ± 0.004	0.036 ± 0.004	0.004 ± 0.003
13	0.056 ± 0.007	0.034 ± 0.007	0.022 ± 0.012	0.055 ± 0.008	0.028 ± 0.008	0.027 ± 0.014	0.050 ± 0.005	0.044 ± 0.005	0.006 ± 0.005
15	0.063 ± 0.009	0.032 ± 0.009	0.030 ± 0.016	0.058 ± 0.006	0.047 ± 0.006	0.011 ± 0.007	0.049 ± 0.005	0.049 ± 0.005	0.001 ± 0.002
17	0.052 ± 0.008	0.024 ± 0.008	0.028 ± 0.015	0.043 ± 0.005	0.033 ± 0.005	0.011 ± 0.007	0.049 ± 0.005	0.045 ± 0.005	0.003 ± 0.003
19	0.048 ± 0.008	0.021 ± 0.008	0.027 ± 0.014	0.049 ± 0.005	0.040 ± 0.005	0.008 ± 0.005	0.046 ± 0.005	0.041 ± 0.005	0.005 ± 0.004
21	0.045 ± 0.005	0.027 ± 0.005	0.018 ± 0.009	0.045 ± 0.003	0.038 ± 0.004	0.007 ± 0.004	0.043 ± 0.004	0.031 ± 0.004	0.012 ± 0.007
ISMER									
9	0.037 ± 0.008	0.008 ± 0.008	0.029 ± 0.014	0.021 ± 0.002	0.017 ± 0.002	0.003 ± 0.002	0.026 ± 0.004	0.012 ± 0.004	0.013 ± 0.007
11	0.050 ± 0.008	0.021 ± 0.008	0.030 ± 0.015	0.027 ± 0.003	0.022 ± 0.003	0.005 ± 0.003	0.036 ± 0.004	0.026 ± 0.004	0.010 ± 0.006
13	0.041 ± 0.009	0.012 ± 0.009	0.029 ± 0.017	0.028 ± 0.003	0.025 ± 0.003	0.003 ± 0.003	0.041 ± 0.005	0.027 ± 0.005	0.014 ± 0.009
15	0.050 ± 0.011	0.014 ± 0.011	0.035 ± 0.020	0.026 ± 0.003	0.023 ± 0.003	0.004 ± 0.003	0.036 ± 0.004	0.027 ± 0.004	0.009 ± 0.006
17	0.050 ± 0.010	0.018 ± 0.010	0.033 ± 0.019	0.038 ± 0.004	0.038 ± 0.004	0.001 ± 0.002	0.036 ± 0.004	0.027 ± 0.004	0.009 ± 0.006
19	0.033 ± 0.006	0.013 ± 0.006	0.020 ± 0.012	0.034 ± 0.003	0.030 ± 0.004	0.003 ± 0.003	0.028 ± 0.004	0.016 ± 0.004	0.012 ± 0.007
21	0.024 ± 0.003	0.012 ± 0.004	0.012 ± 0.007	0.025 ± 0.002	0.022 ± 0.002	0.003 ± 0.002	0.032 ± 0.005	0.016 ± 0.005	0.016 ± 0.009

Table S2 Raw data of body mass. Body mass measurement (g) for domestic, Laval, and Rupert fish for each family, each age, and each environment (running freshwater, seasonal temperature variations [ISMER]; recirculating water, constant 10° temperature conditions [LARSA]). Pedigree information is given by the Sir and Dam identification.

Table S2 is available for download at http://www.g3journal.org/lookup/suppl/doi:10.1534/g3.112.005017/-/DC1.

Table S3 Body mass of breeders. Body mass measurements (g) of sirs and dams used to make the different Domestic, Laval, and Rupert full-sib families.

		Sir		Dam	
Population	Family	ID	Body mass	ID	body mass
domestic	D1	Sd1	950	Dd1	720
domestic	D2	Sd2	640	Dd2	760
domestic	D3	Sd3	670	Dd3	640
domestic	D4	Sd4	850	Dd4	680
domestic	D5	Sd5	790	Dd5	680
domestic	D6	Sd6	820	Dd6	720
domestic	D7	Sd7	810	Dd7	660
domestic	D8	Sd8	840	Dd8	620
domestic	D9	Sd9	910	Dd9	750
domestic	D10	Sd10	770	Dd10	790
Laval	L1	SL1	1400	DL1	900
Laval	L2	SL2	1100	DL2	1100
Laval	L3	SL3	1500	DL3	950
Laval	L4	SL4	1350	DL4	1200
Laval	L5	SL5	1100	DL5	1400
Laval	L6	SL6	1250	DL6	950
Laval	L7	SL7	1200	DL7	1100
Laval	L8	SL8	1000	DL8	1500
Laval	L9	SL9	1100	DL9	700
Laval	L10	SL10	1500	DL10	900
Rupert	R1	SR1	604	DR1	371
Rupert	R2	SR2	1514	DR2	394
Rupert	R3	SR3	604	DR3	475
Rupert	R4	SR4	638	DR4	428
Rupert	R5	SR5	1184	DR5	719
Rupert	R6	SR6	680	DR6	478
Rupert	R7	SR7	271	DR7	435
Rupert	R8	SR8	804	DR8	419
Rupert	R9	SR9	752	DR9	672
Rupert	R10	SR10	669	DR10	311

Table S4 Environmental rearing conditions. Natural photoperiod and temperature (°) conditions both at ISMER and LARSA (running freshwater, seasonal temperature variations [ISMER]; recirculating water, constant 10° temperature conditions [LARSA]).

				Temperature	
Year	Month	Week	Photoperiod	ISMER	LARSA
2006	September	38	13L:11D	15.0	10
2006	September	39	12.5L:11.5D	14.0	10
2006	October	40	12L:12L	13.0	10
2006	October	41	11.5L:12.5D	13.0	10
2006	October	42	11L:13D	12.0	10
2006	October	43	10.5L:13.5D	11.0	10
2006	November	44	10.5:13.5D	10.0	10
2006	November	45	10L:14D	10.0	10
2006	November	46	9.5L:14.5D	10.0	10
2006	November	47	9.5L:14.5D	9.0	10
2006	November	48	8.5L:15.5D	8.5	10
2006	December	49	8.5L:15.5D	8.0	10
2006	December	50	8.5L:15.5D	7.0	10
2006	December	51	8.5L:15.5D	6.5	10
2006	December	52	8.5L:15.5D	6.0	10
2007	January	1	8.5L:15.5D	5.0	10
2007	January	2	8.5L:15.5D	5.0	10
2007	January	3	9L:15D	5.0	10
2007	January	4	10L:14D	5.0	10
2007	January	5	10L:14D	4.0	10
2007	February	6	10L:14D	4.0	10
2007	February	7	10.5L:13.5D	3.0	10
2007	February	8	11L:13D	3.0	10
2007	February	9	11L:13D	3.0	10
2007	March	10	11.5L:12.5D	3.0	10
2007	March	11	11.5L:12.5D	3.0	10
2007	March	12	12.5L:11.5D	3.0	10
2007	March	13	12.5L:11.5D	3.0	10
2007	April	14	13L:11D	3.0	10
2007	April	15	13L:11D	3.5	10
2007	April	16	13.5L:10.5D	3.5	10
2007	April	17	14L:10D	3.5	10
2007	April	18	14L:10D	4.5	10
2007	May	19	14.5:9.5D	5.0	10
2007	May	20	14.5L:9.5D	6.5	10

2007	May	21	15L:9D	8.0	10
2007	May	22	15L:9D	9.0	10
2007	June	23	16L:8D	9.5	10
2007	June	24	16L:8D	10.5	10
2007	June	25	16L:8D	11.5	10
2007	June	26	16L:8D	12.0	10
2007	July	27	15.5L:8.5D	12.5	10
2007	July	28	15.5L:8.5D	13.0	10
2007	July	29	15.5L:8.5D	14.0	10
2007	July	30	15.5L:8.5D	14.0	10
2007	July	31	15L:9D	15.0	10
2007	August	32	15L:9D	15.0	10
2007	August	33	14.5L:9.5D	15.0	10
2007	August	34	14.5L:9.5D	15.0	10
2007	August	35	14L:10D	15.0	10
2007	September	36	13L:11D	15.0	10
2007	September	37	13L:11D	15.0	10
2007	September	38	13L:11D	14.0	10
2007	September	39	12.5L:11.5D	14.0	10
2007	October	40	12L:12L	14.0	10
2007	October	41	11.5L:12.5D	13.0	10
2007	October	42	11L:13D	13.0	10
2007	October	43	10.5L:13.5D	12.5	10
2007	November	44	10.5:13.5D	11.0	10
2007	November	45	10L:14D	10.5	10
