# Supplementary material for: Strain Specific Genotype−Environment Interactions and Evolutionary Potential for Body Mass in Brook Charr (Salvelinus fontinalis)
Source: G3 (Bethesda). 2013 Mar 1;3(3):379–86. doi: 10.1534/g3.112.005017 (PMC3583447; doi:10.1534/g3.112.005017)
Supplement: Supporting Information [file supp_3.3.379_TableS1.pdf]

Table S1 Genetic components of body mass at age. Estimates for the three populations at each age and in both environments (running freshwater, seasonal temperature variations [ISMER]; recirculating freshwater, constant 10°C temperature conditions [LARSA]). Estimates of total phenotypic (V_P), residual (V_R), and additive (V_A) variance components; means ± SE.

Age in	domestic			Laval			Rupert		
months	V_P	V_R	V_A	V_P	V_R	V_A	V_P	V_R	V_A
LARSA									
2	0.004 ± 0.001	0.000 ± 0.000	0.004 ± 0.001	0.003 ± 0.001	0.002 ± 0.001	0.002 ± 0.001	0.014 ± 0.002	0.006 ± 0.002	0.008 ± 0.004
4	0.016 ± 0.002	0.010 ± 0.002	0.006 ± 0.003	0.010 ± 0.001	0.008 ± 0.001	0.002 ± 0.001	0.031 ± 0.006	0.006 ± 0.006	0.025 ± 0.012
7	0.029 ± 0.005	0.010 ± 0.005	0.019 ± 0.009	0.017 ± 0.003	0.005 ± 0.003	0.012 ± 0.006	0.026 ± 0.004	0.013 ± 0.004	0.013 ± 0.007
9	0.045 ± 0.010	0.006 ± 0.010	0.039 ± 0.019	0.026 ± 0.004	0.011 ± 0.004	0.015 ± 0.008	0.033 ± 0.005	0.019 ± 0.005	0.014 ± 0.008
11	0.050 ± 0.010	0.011 ± 0.010	0.039 ± 0.019	0.043 ± 0.007	0.019 ± 0.007	0.023 ± 0.012	0.040 ± 0.004	0.036 ± 0.004	0.004 ± 0.003
13	0.056 ± 0.007	0.034 ± 0.007	0.022 ± 0.012	0.055 ± 0.008	0.028 ± 0.008	0.027 ± 0.014	0.050 ± 0.005	0.044 ± 0.005	0.006 ± 0.005
15	0.063 ± 0.009	0.032 ± 0.009	0.030 ± 0.016	0.058 ± 0.006	0.047 ± 0.006	0.011 ± 0.007	0.049 ± 0.005	0.049 ± 0.005	0.001 ± 0.002
17	0.052 ± 0.008	0.024 ± 0.008	0.028 ± 0.015	0.043 ± 0.005	0.033 ± 0.005	0.011 ± 0.007	0.049 ± 0.005	0.045 ± 0.005	0.003 ± 0.003
19	0.048 ± 0.008	0.021 ± 0.008	0.027 ± 0.014	0.049 ± 0.005	0.040 ± 0.005	0.008 ± 0.005	0.046 ± 0.005	0.041 ± 0.005	0.005 ± 0.004
21	0.045 ± 0.005	0.027 ± 0.005	0.018 ± 0.009	0.045 ± 0.003	0.038 ± 0.004	0.007 ± 0.004	0.043 ± 0.004	0.031 ± 0.004	0.012 ± 0.007
ISMER									
9	0.037 ± 0.008	0.008 ± 0.008	0.029 ± 0.014	0.021 ± 0.002	0.017 ± 0.002	0.003 ± 0.002	0.026 ± 0.004	0.012 ± 0.004	0.013 ± 0.007
11	0.050 ± 0.008	0.021 ± 0.008	0.030 ± 0.015	0.027 ± 0.003	0.022 ± 0.003	0.005 ± 0.003	0.036 ± 0.004	0.026 ± 0.004	0.010 ± 0.006
13	0.041 ± 0.009	0.012 ± 0.009	0.029 ± 0.017	0.028 ± 0.003	0.025 ± 0.003	0.003 ± 0.003	0.041 ± 0.005	0.027 ± 0.005	0.014 ± 0.009
15	0.050 ± 0.011	0.014 ± 0.011	0.035 ± 0.020	0.026 ± 0.003	0.023 ± 0.003	0.004 ± 0.003	0.036 ± 0.004	0.027 ± 0.004	0.009 ± 0.006
17	0.050 ± 0.010	0.018 ± 0.010	0.033 ± 0.019	0.038 ± 0.004	0.038 ± 0.004	0.001 ± 0.002	0.036 ± 0.004	0.027 ± 0.004	0.009 ± 0.006
19	0.033 ± 0.006	0.013 ± 0.006	0.020 ± 0.012	0.034 ± 0.003	0.030 ± 0.004	0.003 ± 0.003	0.028 ± 0.004	0.016 ± 0.004	0.012 ± 0.007
21	0.024 ± 0.003	0.012 ± 0.004	0.012 ± 0.007	0.025 ± 0.002	0.022 ± 0.002	0.003 ± 0.002	0.032 ± 0.005	0.016 ± 0.005	0.016 ± 0.009
