# Supplementary material for: Strain Specific Genotype−Environment Interactions and Evolutionary Potential for Body Mass in Brook Charr (Salvelinus fontinalis)
Source: G3 (Bethesda). 2013 Mar 1;3(3):379–86. doi: 10.1534/g3.112.005017 (PMC3583447; doi:10.1534/g3.112.005017)
Supplement: Supporting Information [file supp_3.3.379_TableS3.pdf]

Table S3 Body mass of breeders. Body mass measurements (g) of sirs and dams used to make the different Domestic, Laval, and Rupert full-sib families.

		Sir		Dam	
Population	Family	ID	Body mass	ID	body mass
domestic	D1	Sd1	950	Dd1	720
domestic	D2	Sd2	640	Dd2	760
domestic	D3	Sd3	670	Dd3	640
domestic	D4	Sd4	850	Dd4	680
domestic	D5	Sd5	790	Dd5	680
domestic	D6	Sd6	820	Dd6	720
domestic	D7	Sd7	810	Dd7	660
domestic	D8	Sd8	840	Dd8	620
domestic	D9	Sd9	910	Dd9	750
domestic	D10	Sd10	770	Dd10	790
Laval	L1	SL1	1400	DL1	900
Laval	L2	SL2	1100	DL2	1100
Laval	L3	SL3	1500	DL3	950
Laval	L4	SL4	1350	DL4	1200
Laval	L5	SL5	1100	DL5	1400
Laval	L6	SL6	1250	DL6	950
Laval	L7	SL7	1200	DL7	1100
Laval	L8	SL8	1000	DL8	1500
Laval	L9	SL9	1100	DL9	700
Laval	L10	SL10	1500	DL10	900
Rupert	R1	SR1	604	DR1	371
Rupert	R2	SR2	1514	DR2	394
Rupert	R3	SR3	604	DR3	475
Rupert	R4	SR4	638	DR4	428
Rupert	R5	SR5	1184	DR5	719
Rupert	R6	SR6	680	DR6	478
Rupert	R7	SR7	271	DR7	435
Rupert	R8	SR8	804	DR8	419
Rupert	R9	SR9	752	DR9	672
Rupert	R10	SR10	669	DR10	311
