# Supplementary material for: Strain Specific Genotype−Environment Interactions and Evolutionary Potential for Body Mass in Brook Charr (Salvelinus fontinalis)
Source: G3 (Bethesda). 2013 Mar 1;3(3):379–86. doi: 10.1534/g3.112.005017 (PMC3583447; doi:10.1534/g3.112.005017)
Supplement: Supporting Information [file supp_3.3.379_TableS4.pdf]

Table S4 Environmental rearing conditions. Natural photoperiod and temperature (°) conditions both at ISMER and LARSA (running freshwater, seasonal temperature variations [ISMER]; recirculating water, constant 10° temperature conditions [LARSA]).

				Temperature	
Year	Month	Week	Photoperiod	ISMER	LARSA
2006	September	38	13L:11D	15.0	10
2006	September	39	12.5L:11.5D	14.0	10
2006	October	40	12L:12L	13.0	10
2006	October	41	11.5L:12.5D	13.0	10
2006	October	42	11L:13D	12.0	10
2006	October	43	10.5L:13.5D	11.0	10
2006	November	44	10.5:13.5D	10.0	10
2006	November	45	10L:14D	10.0	10
2006	November	46	9.5L:14.5D	10.0	10
2006	November	47	9.5L:14.5D	9.0	10
2006	November	48	8.5L:15.5D	8.5	10
2006	December	49	8.5L:15.5D	8.0	10
2006	December	50	8.5L:15.5D	7.0	10
2006	December	51	8.5L:15.5D	6.5	10
2006	December	52	8.5L:15.5D	6.0	10
2007	January	1	8.5L:15.5D	5.0	10
2007	January	2	8.5L:15.5D	5.0	10
2007	January	3	9L:15D	5.0	10
2007	January	4	10L:14D	5.0	10
2007	January	5	10L:14D	4.0	10
2007	February	6	10L:14D	4.0	10
2007	February	7	10.5L:13.5D	3.0	10
2007	February	8	11L:13D	3.0	10
2007	February	9	11L:13D	3.0	10
2007	March	10	11.5L:12.5D	3.0	10
2007	March	11	11.5L:12.5D	3.0	10
2007	March	12	12.5L:11.5D	3.0	10
2007	March	13	12.5L:11.5D	3.0	10
2007	April	14	13L:11D	3.0	10
2007	April	15	13L:11D	3.5	10
2007	April	16	13.5L:10.5D	3.5	10
2007	April	17	14L:10D	3.5	10
2007	April	18	14L:10D	4.5	10
2007	May	19	14.5:9.5D	5.0	10
2007	May	20	14.5L:9.5D	6.5	10

2007	May	21	15L:9D	8.0	10
2007	May	22	15L:9D	9.0	10
2007	June	23	16L:8D	9.5	10
2007	June	24	16L:8D	10.5	10
2007	June	25	16L:8D	11.5	10
2007	June	26	16L:8D	12.0	10
2007	July	27	15.5L:8.5D	12.5	10
2007	July	28	15.5L:8.5D	13.0	10
2007	July	29	15.5L:8.5D	14.0	10
2007	July	30	15.5L:8.5D	14.0	10
2007	July	31	15L:9D	15.0	10
2007	August	32	15L:9D	15.0	10
2007	August	33	14.5L:9.5D	15.0	10
2007	August	34	14.5L:9.5D	15.0	10
2007	August	35	14L:10D	15.0	10
2007	September	36	13L:11D	15.0	10
2007	September	37	13L:11D	15.0	10
2007	September	38	13L:11D	14.0	10
2007	September	39	12.5L:11.5D	14.0	10
2007	October	40	12L:12L	14.0	10
2007	October	41	11.5L:12.5D	13.0	10
2007	October	42	11L:13D	13.0	10
2007	October	43	10.5L:13.5D	12.5	10
2007	November	44	10.5:13.5D	11.0	10
2007	November	45	10L:14D	10.5	10
